# Application of Boltzmann kinetic equations to model X-ray-created warm dense matter and plasma

**DOI:** 10.1098/rsta.2022.0216

**Published:** 2023-08-21

**Authors:** Beata Ziaja, John Jasper Bekx, Martin Masek, Nikita Medvedev, Vladimir Lipp, Vikrant Saxena, Michal Stransky

**Affiliations:** ^1^ Center for Free-Electron Science CFEL, Deutsches Elektronen-Synchrotron DESY, Notkestr. 85, 22607 Hamburg, Germany; ^2^ Institute of Nuclear Physics, Polish Academy of Sciences, Radzikowskiego 152, 31-342 Krakow, Poland; ^3^ Institute of Physics, Czech Academy of Sciences, Na Slovance 2,182 21 Prague 8, Czech Republic; ^4^ Institute of Plasma Physics, Czech Academy of Sciences, Za Slovankou 3, 182 00 Prague 8, Czech Republic; ^5^ Department of Physics, Indian Institute of Technology Delhi,New Delhi 110016, India; ^6^ European XFEL, Holzkoppel 4, 22869 Schenefeld, Germany

**Keywords:** X-ray free-electron lasers, warm dense matter, plasma, Boltzmann kinetic equations

## Abstract

In this review, we describe the application of Boltzmann kinetic equations for modelling warm dense matter and plasma formed after irradiation of solid materials with intense femtosecond X-ray pulses. Classical Boltzmann kinetic equations are derived from the reduced N-particle Liouville equations. They include only single-particle densities of ions and free electrons present in the sample. The first version of the Boltzmann kinetic equation solver was completed in 2006. It could model non-equilibrium evolution of X-ray-irradiated finite-size atomic systems. In 2016, the code was adapted to study plasma created from X-ray-irradiated materials. Additional extension of the code was then also performed, enabling simulations in the hard X-ray irradiation regime. In order to avoid treatment of a very high number of active atomic configurations involved in the excitation and relaxation of X-ray-irradiated materials, an approach called ‘predominant excitation and relaxation path’ (PERP) was introduced. It limited the number of active atomic configurations by following the sample evolution only along most PERPs. The performance of the Boltzmann code is illustrated in the examples of X-ray-heated solid carbon and gold. Actual model limitations and further model developments are discussed.

This article is part of the theme issue 'Dynamic and transient processes in warm dense matter'.

## 1. Introduction

Already for two decades the X-ray free-electron lasers (XFELs), e.g. [1–6], have enabled a unique insight into previously unavailable and still not fully explored regime of ultrafast processes occurring in matter on femtosecond time scales. Intense X-ray pulses from XFELs may serve as a probe, providing access to the information on transient electronic and structural transitions in matter, acquired, among others, with the powerful technique of coherent diffraction imaging. The X-rays may also act as a pump, triggering strong excitation of electronic sub-system on femtosecond time scales. This brings the matter into a strongly non-equilibrium regime, e.g. [7–9], in particular into the transient warm dense matter (WDM) state, e.g. [10–12]. WDM is the state of matter at the frontier between a plasma and a condensed phase. It is key to astrophysics, planetary science and inertial confinement fusion research, but its electronic and ionic structure and dynamics remain poorly understood. Among the methods used to diagnose WDM, spectroscopic methods play a very important role [13–17].

For the analysis of experimental results on X-ray-created WDM and plasma, the development of dedicated theoretical tools, able to describe the evolution of these states under strongly non-equilibrium conditions, is necessary. WDM, especially, represents a modelling challenge, bordering between a plasma and a solid. Among the available modelling methods for WDM are collisional-radiative models (for a recent review, see [18]), superconfiguration approaches (e.g. [19–23]), molecular dynamics methods (e.g. [24–29]), ab-initio methods such as Quantum Monte Carlo (for a recent review, see [30]) or the finite-temperature Hartree–Fock–Slater approach [31], and the density functional theory (also time-dependent) approaches, e.g. [32–35].

We will report here on a robust and computationally efficient plasma simulation tool based on the Boltzmann kinetic equations. The initial development of the Boltzmann model was described in [36–44]. Its performance for WDM created from solid samples was demonstrated on the example of WDM carbon [44] and warm dense gold [45], the latter in comparison with the available experimental data. The Boltzmann model uses atomistic approximation, i.e. it assumes that the simulated sample is initially an assembly of unbound atoms and that for all physical processes, atomic cross-sections and rates are applied. This approximation can be accurately applied to simulate samples irradiated with high-intensity X-rays, where chemical dynamics does not play a significant role. Also, the Boltzmann tool can accurately follow non-equilibrium stages of sample evolution, including complicated atomic excitation and relaxation paths also in heavy materials, which is important for spectroscopic applications, e.g. [17].

We now discuss the features of the Boltzmann model in more detail. As explained above, we assume that the simulated sample initially consists of unbound atoms, and the appearance of ions and free electrons follows as a result of X-ray irradiation. Modelling of ionization dynamics in such a sample can be efficiently performed, using a continuum approach [36,46,47]. Such an approach solves evolution equations on phase-space grid for density distributions of electrons, atoms and ions. This significantly reduces computational cost as it depends then only on the grid size and does not scale quadratically with particle number, O(N^2^), as typical for particle approaches. This enables efficient treatment of large samples. The simulation of the non-equilibrium stage of the sample evolution requires application of full kinetic equations, delivering the information on the transient electron and ion distributions and including atomic configuration active during the excitation and relaxation of the irradiated sample.

Another modelling difficulty is that energetic X-rays can release inner-shell electrons, leaving core holes behind. The holes can relax along complex pathways, especially in heavier elements. This relaxation also involves collisional processes and a large number of active atomic configurations. Their very high number can make the respective set of evolution equations (including each configuration) practically insolvable. For example, the total number of atomic configurations in carbon (*Z* = 6) is 27, where *Z* is the atomic number. The corresponding set of evolution equations can then be easily solved for all the configurations. The same is valid for other light elements. But this is already not the case for noble gases, neon (*Z* = 10) and argon (*X* = 18), where the total numbers of atomic configurations are 63 and 1323, respectively. The rapid increase of the atomic configuration number as a function of Z limits the application of kinetic simulations to low-*Z* materials. In order to avoid this problem, superconfiguration approaches, such as, e.g. [19–23,48] were introduced. They do not treat individual atomic configurations but use instead sets of ‘averaged’ configurations [49,50]. For spectroscopic applications, a virtual contour shape kinetic method has been proposed in [51].

In the Boltzmann model, an alternative approach is used. It was first proposed in [44]. Therein, in order to avoid the treatment of a very high number of active atomic configurations involved in the excitation and relaxation of the X-ray-irradiated materials, an approach called ‘predominant excitation and relaxation path’ (PERP) was introduced. It limited the number of active atomic configurations by following the sample excitation and relaxation only along the most probable relaxation paths (including predominant collisional processes). That scheme enabled computationally efficient simulations of the complicated pathways of atomic excitation and relaxation in heavy materials, such as gold [45] and copper [17].

## 2. Kinetic equation approach to model X-ray-irradiated samples

### (a) Physical picture

Classical Boltzmann kinetic equations are derived from the reduced N-particle Liouville equations. They follow the evolution of single-particle densities in phase-space. We adapted them to model X-ray-irradiated solid or plasma systems, assuming that these samples are built of ions in various atomic configurations and of free electrons (atomistic approximation). Each of these constituents is represented by a classical **phase-space** density. The resulting set of kinetic equations for the electron distribution in phase-space, *ρ*^(*e*)^, and for distributions of various atomic configurations, *ρ*^(*i,j*)^, is

2.1 $$\partial_{t}\rho^{\left(e\right)}(\text{r},\text{v},t)+\text{v}\cdot \partial_{\text{r}}\rho^{\left(e\right)}(\text{r},\text{v},t)-\frac{{\text{F}}_{\text{EM}}(\text{r},\text{v},t)}{m}\cdot \partial_{\text{v}}\rho^{\left(e\right)}(\text{r},\text{v},t)=\Omega^{\left(e\right)}(\rho^{\left(e\right)},\rho^{\left(i,j\right)},\text{r},\text{v},t)$$

and

2.2 $$\partial_{t}\rho^{\left(i,j\right)}(\text{r},\text{v},t)+\text{v}\cdot \partial_{\text{r}}\rho^{\left(i,j\right)}(\text{r},\text{v},t)+i\cdot \frac{{\text{F}}_{\text{EM}}(\text{r},\text{v},t)}{M}\cdot \partial_{\text{v}}\rho^{\left(i,j\right)}(\text{r},\text{v},t)=\Omega^{\left(i,j\right)}(\rho^{\left(e\right)},\rho^{\left(i,j\right)},\text{r},\text{v},t).$$

The index, *i* = 0, … , *N_J_*, denotes the ion charge (with *N_J_* being the highest charge state in the system), and the index, *j* = 0, … , *N_C_(i)*, denotes the active configuration number (with *N_C_(i)* being the maximal number of ion configurations considered for a fixed *i*th ion charge). Electron and ion masses are *m* and *M,* respectively.

In the case of X-ray irradiation, the magnetic component of the electromagnetic force, **F**_EM_, in equations ((2.1)–(2.2)) can be neglected, as well as the interaction of the X-ray laser field with charged particles in the sample (for more details, see [36]). This reduces the force to the component representing electrostatic interactions between charges in the sample which is a non-local function of electron and ion densities (see Eq. (5) in [36]).

Initial conditions for equations (2.1)–(2.2) are given by a neutral atom distribution function. The free-electron distribution is equal to zero. During the non-equilibrium evolution of X-ray-irradiated samples, the emerging free-electron and ion densities change: (i) due to photoinduced processes such as photoexcitation and Auger decay and (ii) due to the electronic collisional processes including elastic electron–ion collision, electron impact ionization and three-body recombination. These effects are modelled with the collision terms, *Ω*^(e)^ and *Ω*^(i,j)^. They describe the respective changes of transient electron and ion densities due to the above processes and also due to the short-range electron–electron scattering [52]. The respective rates and cross-sections are derived in the atomistic approximation, i.e. assuming here the interaction of an isolated atom with an impact particle. They are included in two- and three-body Boltzmann collision integrals in the equations (for details see, e.g. [52,53]). The rates and cross-sections of atomic processes induced by X-ray photons were calculated with the XATOM code [27,54]. The collisional ionization cross-sections and the respective recombination rates were obtained from the Lotz formulae [55]. The short-range electron–electron scattering was modelled, using the Fokker–Planck collision integral [52]. As the electron system is treated as a classical one in this model, the Pauli blocking effect is not included. More details can be found in [36]. Please note that if the collision terms are put equal to zero, the Boltzmann equations, equations (2.1)–(2.2), reduce to the Vlasov equations [52], which follow the evolution of a collisionless plasma.

The terms on the left-hand side and right-hand side of the equations, equations (2.1)–(2.2), *per construction* conserve the particle number and the total energy in the system. This applies also for the numerical algorithms applied to solve the kinetic equations [36]. It was checked by dedicated tests in all numerical implementations of the Boltzmann equations performed so far.

The first version of the Boltzmann equation solver was described in [36] and applied there to study noble-gas clusters irradiated with VUV/XUV radiation. Kinetic equations applied included only ground state ion configurations because the incoming XUV photons could excite electrons only from valence levels. The inverse bremsstrahlung process contributed to the free-electron heating in this photon energy regime. The electron recombination could be neglected, as due to inverse bremsstrahlung heating, the electron temperature was much too high for this process to significantly contribute. Further code applications to noble gas systems followed, with ionic time-of-flight spectra studied there. They were presented in [37–39].

In [40], the modelling of three-body recombination was added to the code, extending its applicability both to longer simulation time scales and to higher radiation intensities. In [41], the analysis of the electron spectra was for the first time performed with the Boltzmann equations, without any ‘instantaneous thermalization’ assumption, frequently applied at that time (e.g. [56]). The first application of the code to model non-equilibrium finite-size WDM and plasma was performed in [42]. Femtosecond thermalization of electrons created after X-ray irradiation of hydrogen was studied there. Also heterogeneous samples, consisting of different elements, were studied with the Boltzmann code. In [43], for the first time atomic clusters containing atoms of two noble gases, could be successfully treated.

Later, in [44], the Boltzmann equation solver was modified to enable studies of WDM and plasma created from bulk materials irradiated by X-rays. High-intensity X-ray pulses can trigger spatially homogeneous ionization dynamics within a large volume inside the irradiated material. The necessary conditions are wide beam focusing and the thickness of the target layer comparable with the penetration depth of the X-ray photons [57]. The homogeneous ionization dynamics imply that both electron and ion distributions can be assumed to be spatially uniform. Their evolution then becomes **r**-independent:

2.3 $$\begin{array}{l} \partial_{\text{r}}\rho^{\left(e\right)}(\text{r},\text{v},t)=0\\ \partial_{\text{r}}\rho^{\left(i,j\right)}(\text{r},\text{v},t)=0. \end{array}$$

The direct consequence of the spatial charge homogeneity is the quasineutrality within the sample: at each space point, the electron and gross ion charges are identical. As the net charge is equal to zero at each spatial point, this makes the component of the electrostatic force representing the mutual interactions of electrons and ions equal to zero as well. The modified equations still treat fast-electron thermalization, as the short-range electron–electron interaction term is unaffected by equations (2.3).

Numerical solving of the Boltzmann equations is significantly faster with these simplifications, when comparing with the case of finite samples, as no adaptive stability condition [53,58] is then needed due to the lack of real-space partial derivatives of particle distributions in the equations, cf. equations (2.3). This enables computationally efficient simulations of ionization dynamics within X-ray-irradiated bulk material. For more details, see [44].

Another extension of the code was performed, enabling treatment of hard X-ray irradiation. In order to avoid the bottleneck of very high number of active atomic configurations involved in the excitation and relaxation of X-ray-irradiated materials, an approach called PERP was introduced in [44]. It limited the number of active atomic configurations by following the sample relaxation along the most probable relaxation paths (including also collisional relaxation processes). Their choice was determined by the largest cross-sections and transitions rates. For example, the simplest PERP scheme includes only atomic configurations present in the excitation and relaxation paths with the most probable photoionization processes and the most probable Auger decays and the predominant collisional ionization processes (from/to the valence shell of all considered atoms and ions). The accuracy of the scheme was tested in [44] for carbon by performing PERP calculations and comparing them with the results obtained with a calculation including all relaxation paths. Further successful benchmarking, using a molecular dynamics code, was made later [28].

The choice of the PERPs for an X-ray-irradiated material is now performed automatically at the specified pulse conditions by a dedicated script—before the simulation starts. The script allows to apply the approach also to heavy elements (after including radiative decays of core holes), and practically at all X-ray photon energies, available at the currently operating XFEL facilities (less than 30 keV).

### (b) Numerical implementation

Equations (2.1), (2.2) are integro-differential equations, in the most general case, in six-dimensional phase-space. Therefore, only their numerical solution is possible. A significant simplification of the kinetic equations can be achieved: (i) if considering samples with a symmetry and (ii) if applying the angular moment expansion for the electron and ion densities [52,59,60]. In the latter case, the isotropic components of the electron and ion phase-space density distributions should be predominant. This assumption is valid in systems, where there is strong energy dissipation by collisions. This implies that the velocity component of collective transport is small. Applying in addition the condition, equations (2.3), reduces the number of dimensions ultimately to one (the magnitude of the velocity, **|v|**).

The spatially uniform Boltzmann equations for a bulk material are then solved on a grid in velocity space, applying dedicated numerical methods [58,61,62]. The pseudospectral method [62] is used to efficiently evaluate integrals and partial derivatives in the Boltzmann equations. The solver has been carefully tested to check its numerical accuracy. As mentioned earlier, it is conservative in respect to the particle number and total energy, if the source terms are set equal to zero [36]. The code is computationally efficient and can be parallelized. At typical XFEL parameters, a run on a single CPU core takes between a few hours and a few days.

## 3. Performance of the kinetic Boltzmann equations model for X-ray-heated carbon and gold

In [44], we tested the PERP concept for a light element, carbon (*Z* = 6). Carbon bulk of diamond density was irradiated there with (i) a soft X-ray pulse of *E*_photon _= 1000 eV and of maximal intensity of 10^16^ W cm^−2^ and (ii) hard X-ray pulse of *E*_photon _= 5000 eV and maximal intensity of 10^18^ W cm^−2^. Using our Boltzmann solver, we checked that both the average charge and average energy absorbed per atom predicted with full calculation (including all 27 atomic configurations) and with the PERP scheme were in a good agreement.

In [45], we applied our code to a heavy element, gold (*Z* = 79). It was chosen because many theoretical and experimental data from the WDM regime of gold exist (e.g. [63]). The adapted code (with the PERP approach inside) could treat charges up to +9, including 144 various ion configurations of Au. The number of photoinduced transitions between those configurations was 140. For the study, we applied a set of pulse parameters available at the FLASH experimental facility [2] for high-energy-density experiments (photon energy of 245 eV and the FWHM pulse duration of 60 fs). The X-ray pulse irradiated a 30 nm thick layer of Au. The thickness of the Au layer is similar to the attenuation length of 245 eV photons in gold (approx. 37 nm). This prevents the creation of strong gradients for X-ray energy absorbed within the layer. Pulse energy yielded the deposited dose typical for WDM experiments, i.e. 1–2 MJ kg^−1^.

The 245 eV X-ray photons predominantly excited 4f-shell electrons. The 4 f holes left had a lifetime of approximately 6.6 fs. They were filled with electrons, mostly from 5d shell, and 5d Auger electrons were then emitted. Further photoionization of the 4 f core hole state to a double core hole state was also occurring. In parallel to photoinduced transitions, the collisional processes induced by released electrons began to contribute to the ionization dynamics as well. This rapidly increased the average ion charge and led to a fast energy redistribution within the electronic system. A local thermodynamic equilibration of the electronic system followed on tens of femtoseconds time scale.

As mentioned above, the set of possible PERPs prepared for this simulation could follow the evolution of various atomic configurations of charges up to +9. However, for the above specified X-ray pulse parameters, creation of charge states only up to +4 was energetically possible, and therefore, effectively, only a small part of the prepared PERP set was used. The ground state configuration (with delocalized 6s electrons) was built from Au_ +1_ ions. Within the PERP approximation, the number of active configuration for Au_ +2_ ions was 2, and for Au _+3_ and Au_ +4_ ions was 3. Eight photo-induced transitions were involved.

**Figure 1.**
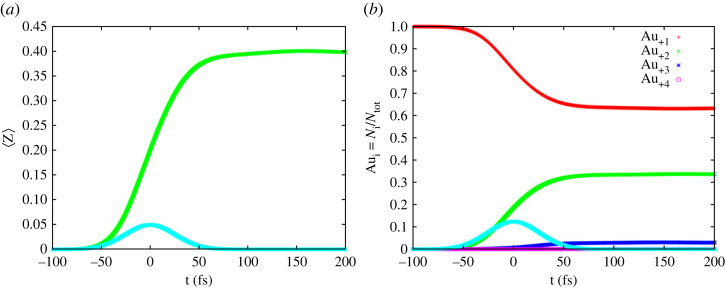
Average charge per atom (*a*) and relative ion content (*b*) in X-ray-irradiated Au layer as a function of time. The deposited X-ray dose was 1.2 MJ kg^−1^. Temporal pulse profile is depicted as well (light blue curve). The X-ray photon energy was 245 eV and the FWHM pulse duration of 60 fs. The peak X-ray intensity at time zero was 2.5 × 10^12^ W cm^−2^.

Figure 1 shows the average ionization degree of the bulk gold (per atom) (figure 1*a*) and the relative charge content, i.e. the number of specific Au ions divided by the initial total number of Au atoms (figure 1*b*), both as a function of time, predicted for the deposited dose of 1.2 MJ kg^−1^. Please recall that in the framework of our atomistic model, the ‘neutral’ bulk contains only ions, Au _+1_. They are not used for the calculation of 〈Z〉. The predictions are shown up to 200 fs after the maximum of the XFEL pulse.

The average charge 〈Z〉 per atom strongly grows after the exposure start (figure 1*a*). It almost stops to change at approximately 100 fs and increases later only slightly. The latter effect is due to the long-time scale Auger decays. Also, three-body recombination starts to play a role at later times, increasing the complexity of the relaxation dynamics. The relative ion content can be seen in figure 1*b*. It is the largest for Au_ +1_ ions (with one delocalized 6s electron) forming neutral bulk Au. The Au_ +2_ charge states are formed from Au_+1_ as a result of a core hole excitation 4f or 5p, or after a photo- or impact ionization from level 5d. The ions Au_+3_ are only a small fraction of the overall number of ions. They can be created through the Auger decay of a core hole (here 4f → 5d 5d), or through a photoionization or an impact ionization of an Au_ +2_ ion. Creation of Au_ +4_ ions follows a similar pathway.

**Figure 2.**
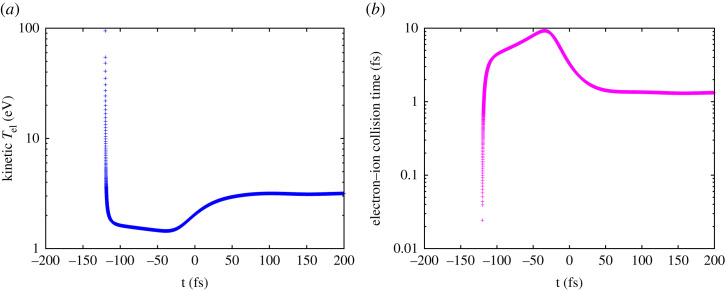
Kinetic electron temperature (*a*) and transient electron–ion collision time (*b*) as a function of time. The deposited dose was 1.2 MJ kg^−1^. The maximum of the FEL pulse intensity is at *t* = 0 fs. The X-ray photon energy was 245 eV and the FWHM pulse duration of 60 fs. The peak X-ray intensity at time zero was 2.5 × 10^12^ W cm^−2^.

Figure 2 shows the predicted kinetic electron temperature and transient electron–ion collision time, the latter calculated self-consistently, using the actual electron–ion collision rates, and the transient electron and ion distributions in the sample. The kinetic electron temperature is calculated as 2/3 of the total kinetic energy of all free electrons above the 6s level (calculated with respect to the 6s level) divided by the number of free electrons above the 6s level. After the electrons thermalize, the kinetic temperature becomes the Maxwell–Boltzmann temperature. The contribution from the delocalized 6s electrons to the kinetic temperature is neglected within the framework of this model, as it constitutes only a small part of the total electron kinetic energy.

Initially, the kinetic electron temperature equals the temperature of the emitted photoelectrons (approx. 93 eV) after the excitation of a 4f level (figure 2*a*). Fast electron thermalization process (through electron cascading) leads to a fast drop of the kinetic temperature at −125 fs. It then shows a plateau until −50 fs. After that time the kinetic temperature slightly increases. This increase is due to the contribution of electrons from later photoionization events and Auger decays. Fast energy exchange among numerous secondary electrons leads to the fast thermalization of the electron distribution already at approximately 50 fs. It is then when kinetic temperature almost stops to change. The ongoing and then completed thermalization is indicated by the electron–ion collision time predicted. Here, this collision time is defined as an inverse of the total electron–ion collision rate calculated per one electron. It was derived in the same way as described in §2.3 of [48]. This calculation takes into account all free electron–bound electron collisions. Initially, the collision time rapidly increases, mimicking the decrease of the kinetic temperature. It reaches a maximum at around −35 fs. It is the same time at which the kinetic *T*_el_ reaches its minimum. After the thermalization of free electrons is completed, it saturates at the value of approximately 1.2 fs. This value is in very good agreement with the equilibrium temperature value measured in [63] after the relaxation of optically excited Au.

**Figure 3.**
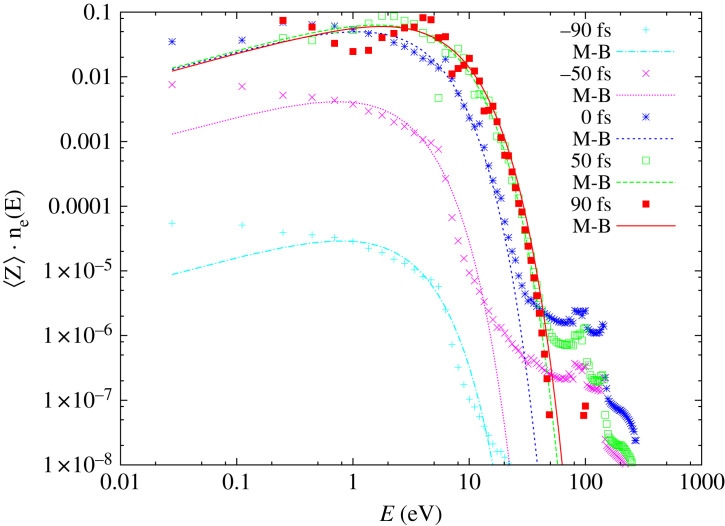
Snapshots of transient electron-energy distributions, 〈Z〉 · n_e_(E), predicted at various time instants (plotted with points), compared with the corresponding Maxwell–Boltzmann distributions (plotted with solid lines of the same colour as the points) obtained with the transient values of electron density and kinetic temperature. Parameters of the X-ray pulse are the same as in figures 1 and 2.

Figure 3 shows the transient free-electron-energy distribution, n_e_ (E) (normalized per electron) as a function of energy. The snapshots are presented for the times: −90 fs, −50 fs, 0 fs (FEL pulse maximum), 50 fs and 90 fs. The normalized free-electron-energy distribution is multiplied by the actual value of the transient ionization degree, 〈Z〉, i.e. the number of free electrons above the 6s level divided by the total number of atoms (figure 1*a*), in order to demonstrate the growth of the free-electron density per atom in the sample. Each snapshot of the transient electron-energy distribution is compared with the corresponding Maxwell–Boltzmann (M–B) distribution obtained using **the actual value of kinetic temperature, electron density and multiplied by the actual value of the transient ionization degree predicted by the Boltzmann model.** Initially, the transient electron-energy distributions are distant from the M–B distributions. This occurs both (i) in the low-energy regime, where the contribution of low-energy thermalized electrons is not yet complete due to the time needed for finishing electron cascading processes and (ii) in the high-energy regime, where the contribution of photo- and Auger electron shows up (spectral peaks at approx. 100 eV). This indicates that the system has not yet thermalized as the ongoing production of high-energy electrons through photoionization and Auger processes prevent this. Some time after time zero, the non-thermal contribution of photo- and Auger electrons almost disappears, and both the high- and the low-energy parts of the transient spectra approach the M–B distribution. This is the time when the majority of high-energy electrons have been already produced and thermalized (for 100 eV impact electrons in Au thermalization time is less than 10 fs). The M–B curves estimated for times 50 fs and 90 fs are very close to each other, and to the respective transient free-electron-energy distributions. This confirms that the Maxwell–Boltzmann thermalization has been achieved, and the electronic system properties could be accurately described, assuming a respective Maxwell–Boltzmann electron-energy distribution. It should be emphasized that the above discussed thermalization time scale predictions were obtained for a specific irradiation case. Generally, temporal scales of the thermalization can be very different, depending on the X-ray photon energy and X-ray pulse fluence. The latter speeds up electron thermalization, as the density of created electrons is then higher, and the rates for short-range energy exchange between electrons increase respectively. Higher photon energies have an opposite effect, as the thermalization of high-energy electrons released by these photons takes longer times.

The predictions on free-electron density and electronic temperature can also be used to evaluate the transient optical properties of WDM Au. For example, using Drude model, we calculated the DC conductivity, *σ*_0_, as a function of time, $\sigma_{0}=\omega_{p}^{\;2}\;\tau /4\pi$ (not shown). There, *ω*_p_ was the electron plasma frequency, and *τ* was the electron–ion collision time. The predicted DC conductivity decreased until free electrons thermalize (approx. 50 fs) (Figure 2), reaching an equilibrium value of 2.7 × 10^16^ 1 s^−1^—in good agreement with the equilibrium value of approximately 3 × 10^16^ 1 s^−1^ measured in [63].

## 4. Further model developments

### (a) Electron–ion interaction and picosecond evolution times

The Boltzmann equation solver was constructed to describe electron and ion dynamics on maximally a few 100 fs time scale. Due to the large ion-mass-to-electron-mass ratio, the ion recoil during collisions between electron and ions could be neglected. The ions remained ‘cold’ during the entire simulation.

However, the electron–ion energy exchange and the mutual thermalization of the electron and ion systems have to be treated, when moving to picosecond simulation time scales. Therefore, the original Boltzmann equation solver was extended for the electron–ion coupling, which has been so far approximated with the Spitzer rate [52]. As in bulk Au the electron–ion coupling is weak, it did not much affect the sample evolution on picosecond time scales. The description of the electron–ion coupling can be further improved, using the dynamical coupling rates from, e.g. [64], available for a variety of materials.

It should be also emphasized that the atomistic plasma code per construction cannot treat band structure. The sample is defined as an ensemble of unbound atoms in this approximation. The interaction of the atoms with X-ray photons and secondary electrons is modelled with atomistic cross-sections and rates. This feature cannot be easily improved, besides including bond energy in the simulations.

### (b) Ionization potential lowering

When a solid sample is irradiated with a high-intensity X-ray pulse, numerous electrons and ions are produced. The ions within the dense plasma formed cannot be any longer modelled as isolated particles due to the plasma environment. For the description of the dense plasma effect on the ionization potential (IP) of the plasma-embedded ions, dedicated modelling [65] is required. For the Boltzmann code, the IP lowering can be estimated with the code XATOM for plasmas of various electron densities and temperatures [27,66]. However, the code is prepared for any implementation of the IP shift, either obtained from phenomenological parametrizations [65] or from ab-initio approaches, e.g. [66]. The value of the shift can be changed on-the-fly, as a function of actual plasma parameters.

### (c) Impact ionization cross-sections

In order to include only predominant processes during sample excitation and relaxation, we consistently treated in the code only the predominant collisional ionization processes, i.e. collisional ionization and recombination from/to the outermost shell of all considered atoms and ions. The latter assumption holds for impact electrons of energy less than 1000 eV. However, at higher impact electron energies, collisional core hole excitations become increasingly probable. They have to be taken into account, when extending the application of the Boltzmann code to X-ray irradiation regimes with photons of a few keV up to several keV energies. This is planned as a future code development.

### (d) Fermi–Dirac statistics

The Boltzmann kinetic equations presented above are classical kinetic equations, with energetic electrons evolving towards Maxwell–Boltzmann distribution. However, one could, in principle, account in the equations also for Pauli blocking [67–69], after introducing discrete free-electron levels. During electron thermalization, this would push electron distribution towards Fermi–Dirac distribution. Such an extension of the code is non-trivial but feasible. It is also planned as a future development.

## 5. Conclusion and outlook

We have described the implementation of our Boltzmann kinetic model to simulate WDM and plasma created from X-ray-irradiated bulk systems. It was illustrated in detail on the example of X-ray-created WDM gold, also analysed in [45]. The Boltzmann model predictions were compared with the available experimental data and found to be in good agreement with them. This encouraging finding has stimulated further applications of the Boltzmann solver. The respective projects on X-ray-heated copper and silicon are ongoing.

With further model developments listed in §4, there is a clear prospect to construct a comprehensive, versatile simulation tool for X-ray-irradiated bulk solids, of computational efficiency much higher than that of typical Molecular Dynamics approaches. This Boltzmann model extension is underway. Its predictions will undergo validation by dedicated experiments.

## Data Availability

This article has no additional data.
